# Anemia in heart failure: evidence from a three-year cross-sectional study in Sudan

**DOI:** 10.1186/s40780-025-00511-9

**Published:** 2025-11-18

**Authors:** Maram M. Elamin, Rayan Hafiz Mohamed, Mohamed Alzaki Ahmed, Mugahed Ahmed Abdullah, Afraah Altahir Abdalrahim, Gheida Alamin Elbushra, Yousif B. Hamdalneel, Kannan O. Ahmed

**Affiliations:** 1 Department of Clinical Pharmacy and Pharmacy Practice, Faculty of Pharmacy, University of Gezira, Wad Medani, Sudan; 2 Department of Pharmacy Practice, College of Pharmacy, National University of Science and Technology, Muscat, Oman

**Keywords:** Anemia, Heart failure, Prevalence, Hospitalization, Sudan

## Abstract

**Background:**

Anemia is a frequent complication of heart failure (HF) that exacerbates cardiac dysfunction and worsens prognosis. However, its exact burden and treatment patterns in Sudan HF remain unknown. Thus, this study aimed to describe the prevalence, characteristics, and management of anemia among Sudanese HF patients.

**Methods:**

We conducted a retrospective cross-sectional study of adult HF patients admitted to Wad Medani Heart Center, Sudan, over three years. Baseline hemoglobin (Hb) was defined as the first measurement within 24–48 h of index admission. Only patients with evaluable baseline Hb were included in the analytic cohort.

**Results:**

Of 557 HF patients, 266 (47.8% of full cohort; 51% of analytic cohort) were anemic (mean Hb 10.68 ± 1.51 g/dL). The mean age was 60.4 ± 18 SD, 136 (51%) were females, 114 (43%) aged more than 65 years, and 77 (28%) had prior HF admissions. Hypertension 136 (51%), diabetes 91 (34%) and chronic kidney disease 75 (28%) were other comorbidities. Anemia was most prevalent in the HF with mid-range ejection fraction (HFmrEF) cohort 134 (50.4%), followed by HF with reduced ejection fraction (HFrEF) 71 (26.7%) and HF with a preserved ejection fraction (HFpEF) 61 (22.9%). Among anemic HF patients, only 77 (29%) received anemia management. Of those, 28(36.4%) received blood transfusions, and 34 (44.2%) received iron supplementation.

**Conclusions:**

Nealy half of HF patients were anemic, particularly older and those with HFmrEF, and treatment was suboptimal. Incorporating routine anemia screening and standardized management into HF care protocols is essential to enhance clinical outcomes.

## Background

Heart failure (HF) is a clinical condition characterized by an inability of the heart to perform cardiovascular function efficiently due to functional and/or structural abnormalities [1]. HF continues to be an important global health problem, with more than 37.7 million worldwide [2]. A common cause of hospitalization is HF; the fatality rate among HF patients keeps going up notably among those who are hospitalized with advanced HF. HF appears in different stages; the New York Heart Association (NYHA) classifies HF into four functional classes: NYHA I to NYHA IV, describing the severity of symptoms and exercise tolerance [3].

Several diseases have been linked to increased morbidity and mortality among HF patients. Among these comorbidities, anemia linked to HF is common, which can cause heart function to deteriorate by activating neuro-hormonal mechanisms [4, 5]. Anemia is defined according to the World Health Organization (WHO), hemoglobin (Hb) levels less than 12 g/dl in females and 13 g/dl in males [4]. Anemia in patients with HF has a complicated etiology with numerous coexisting mechanisms implicated. A relevance between the severity of HF syndrome and the complexity of this etiologic divergence has also been suggested [6]. Inflammation, hemodilution, iron deficiency, erythropoietin levels, prescription medicine, and medullary dysfunction have all been linked to anemia either alone or in combination. Iron deficiency and inflammation are the key pathophysiologic pathways with the most evidence-based medicine data [3, 5, 6].

About one-third of HF patients are affected by anemia, with a prevalence range reported to be 17–70%, depending on HF severity, patients’ demographics, comorbidities, and type of study [2]. Anemia mostly affected the decompensated hospitalized patients, which was reported at 50% of patients and 30% in stable patients [7].

Worldwide reports indicate that the prevalence of anemia in heart failure patients ranges from 34% to 62.6% [8–12]. However, in Sudan, no published study has evaluated the prevalence of anemia among HF patients, particularly in Wad Medani, Gezira State. Therefore, this is the first study aimed at describing the prevalence, characteristics, and management of anemia among Sudanese HF patients at Wad Medani Heart Center, Wad Medani, Gezira State, Central Sudan.

## Methods

### Study setting

The study was conducted at Wad Medani Heart Centre, which is a tertiary hospital located in Wad Medani, Gezira State, Central Sudan. It provides free health care that serves the inhabitants of Gezira State and receives referral patients from neighbouring States within the Central Region. The Medni Heart Centre has 100 beds in three separate fundamental sites of the centre that are composed of the ICU (intensive care unit), the CCU (cardiac care unit), and the HDU (high dependency unit), which also contains wards for both Internal Medicine and Surgery services. In addition, it is equipped with a Cardiac Catheterization Laboratory [13].

### Study design and patient selection

A hospital-based, retrospective cross-sectional descriptive study was conducted by screening the patients’ medical files between January 2019 to June 2021. Patients 18 years and older who were admitted to Medani Heart Center with with the principal diagnosis of HF during study period were included. Patient selection was based on the first hospitalization for HF during the study period, regardless of any prior history of HF, those transferred to other hospitals or had incomplete data were excluded from the study.

### Dada collection

Data were extracted from patients’ medical records using a structured data collection form [14, 15]. These data composed of (1) demographic data for each patient include (age, gender, marital status, education, occupation, and residence), (2) clinical characteristics (primary diagnosis, past medical history), relevant and laboratory investigations (including mainly complete blood count (CBC), haematocrit). Levels of serum ferritin, trans-ferritin saturation, and iron‑binding capacity were also evaluated, and (3) HF medications on admission, along with therapies for anaemia employed.

### Variable definitions

Anaemia was defined as a haemoglobin (Hb) level < 13 g/dl for men and < 12 g/dl for women according to WHO criteria [11, 16]. For the purpose of this study, a HF hospitalization is defined as any documented hospital admission in the patient’s medical record lasting over 24 h due to an exacerbation of HF symptoms. Any admission without clear documentation of HF symptom worsening is excluded. Baseline Hb was defined as the first measurement obtained within 24–48 h of the index admission. Only patients with evaluable baseline Hb were included in the analytic cohort to calculate anaemia prevalence. Repeat admissions for the same patient were analyzed separately for outcomes to avoid misclassification bias.

### Data analysis

Data was analyzed by using the IBM Statistical Package for Social Sciences (SPSS) software package version 25.0 (Armonk, NY: IBM Corp.). Descriptive statistics, including frequencies (percentages), were used to express the qualitative data. A normal distribution was examined by the Kolmogorov‒Smirnov test. Quantitative data that were normally distributed were presented as mean ± standard deviation (SD).

## Results

**Fig. 1:**
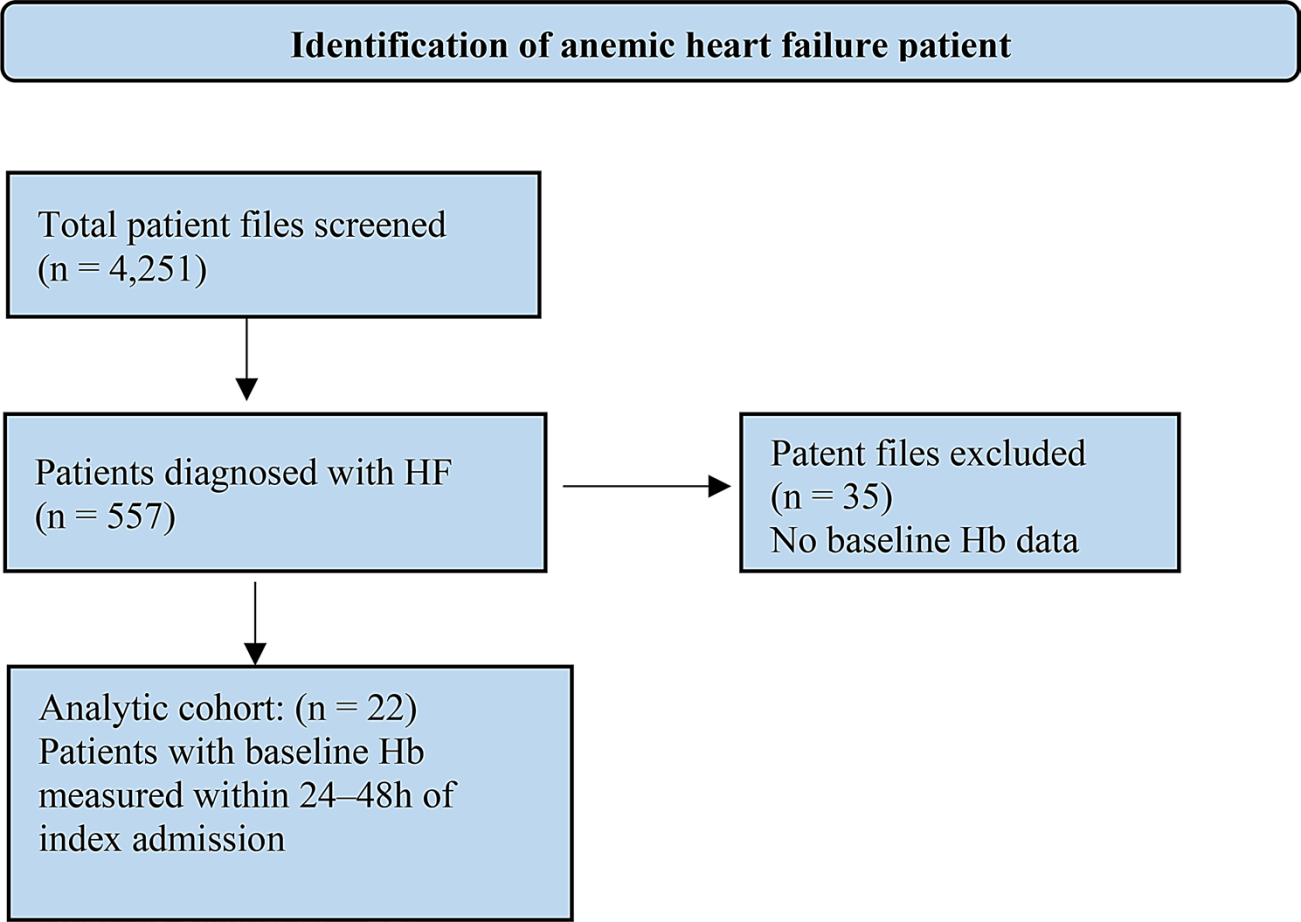
Flow diagram of participant selection process

Over a three-year period, 4,251 patient files were screened, 557 patients were diagnosed with HF. Among all hospitalized HF patients, 266 (47.8%) were anemic. In the analytic cohort with evaluable baseline Hb (*n* = 522), the prevalence of anemia was 51% (Fig. 1).

Among anemic patients,136 (51%) were females, 114 (43%) aged more than 65 years with the mean age of 60.4 years ± 18 SD Table 1. Comorbidities were nearly universal, affecting 260 (98%) of patients; 184 (70%) had 1–3 comorbid diseases, 66 (25%) had 4–5 comorbid diseases, with hypertension 136 (51%) being the highest comorbid condition, followed by diabetes mellitus, 91 (34%), while the least comorbid condition was kidney diseases 75 (28%) as shown in Table 1. On analysis of laboratory and clinical investigations, we found the mean of Hb level was 10.7 ± 1.5 SD, with 73 (27.4%) of patients had an Hb value less than 10 mg/dl, and mean of serum creatinine (Scr) was 1.7 mg/dl ± 2.5 SD. Moreover, the mean of heart rate was 99.9 beat/minute ± 32.8 SD (Table 1). During the study period, HF patients received Angiotensin-converting enzyme inhibitors 56 (21.0%), angiotensin receptor blockers 66 (24.8%), spironolactone 103 (38.7%), furosemide 159 (59.8%), and digoxin 33 (12.4%). Phenotypes of HF were 71(26.7%) HFrEF, 134(50.4%) HFmrEF and 61(22.9%) HFpEF as illustrated in Table 2.

**Table 1:** Socio-Demographics characteristics and comorbidities of anemic HF patients (*n* = 266)

Characteristics	Frequency (%)
**Gender**	
Male	130 (49)
Female	136 (51)
**Age in Years Mean ± SD 60.4 ± 18 SD**	
18–35	35 (13)
36–50	40 (15)
51–65	77 (29)
> 65	114 (43)
**Co-morbidities**	
Yes	260 (98)
No	6 (2)
**Number of co morbid diseases**	
1–3	184 (70)
4–5	66 (25)
> 5	10 (5)
**Number of co morbid diseases** (*n* = 260)	
Hypertension	136(51)
Diabetes mellitus	91(34)
Atrial Fibrillation	80(30)
Kidney disease	75(28)

Percentages calculated for anemic HF patients (*n* = 266). Prevalence of anemia among all HF patients was 47.8% (full cohort) and 51% (analytic cohort). Repeat admissions excluded

**Table 2:** Heart failure subtypes and treatment characteristics (*n* = 266)

Analytic Cohort (*n* = 522)	Frequency (%)
HFrEF (< 40%)	71(26.7)
HFmrEF (40–49%)	134(50.4)
HFpEF (≥ 50%)	61(22.9)
**HF medications**	**Frequency (%)**
Angiotensin Converting Enzyme Inhibitors	56(21)
Angiotensin Receptor Blockers	66 (24.8)
Spironolactone	103 (38.7)
Furosemide	159(59.8)
Digoxin	33(12.4)

Percentages calculated for anemic HF patients (*n* = 266). Repeat admissions excluded

Treatment of anemia in our HF cohort was addressed in 77 patients (29%), with 28 (36.4%) managed exclusively by blood transfusion, 34 (44.2%) receiving sole iron supplementation, and the remaining 15 (19.5%) benefiting from a combined transfusion plus iron-repletion strategy. Within this anemic HF group, 77 patients (28.2%) had a prior HF hospitalization as shown in Table 3.

**Table 3:** Anemia management and hospitalization history (*n* = 266)

Treatment of anaemia	Frequency (%)
Not treated	189 (71.1)
Treated	77 (28.9)
**Types of anaemia Treatment (** ***n*** ** = 77)**	
Blood transfusion only	28 (10.5)
Iron supplementation only	34 (12.8)
Transfusion + Iron supplementation	15(5.6)
**History**	**Frequency (%)**
Never	191 (71.8)
Once	35 (13.2)
Twice	25 (9.4)
≥ 3	15 (5.6)

Percentages calculated for anemic HF patients (*n* = 266). Repeat admissions excluded. Prevalence of anemia among all HF patients reported in Table 1

## Discussion

In this study, the prevalence of anemia among hospitalized HF patients was 47.8% in the full cohort (*n* = 557) and 51% in the analytic cohort with evaluable baseline Hb (*n* = 522), which is lower than several studies carried out in Japan among older patients at 65.7% [17], in Thailand at 62.6% [11], and in Italy at 59% among patients with HF [18]. These higher rates could be linked to factors such as differences in nutritional status, healthcare infrastructure, and comorbid conditions in these populations. However, this findings higher than that reported in England at 33.7% [19], in Swedish at 34% [9], in Italy at 35% among patients with chronic HF, findings from Ethiopia 49.8% [20], and substantially higher than that reported in Senegal 11.6% [18], in a systematic review and meta-analysis from the last decade at 37% [21], in Austria at 17.7% among patients with chronic HF [7], and in Brazzaville, Congo at 42% [12]. These differences might reflect the variations in diagnostic criteria, healthcare access, or the prevalence of underlying factors such as chronic diseases, malnutrition, or socio-economic factors [22–24]. In addition, variations in the anemia definition, screening protocols, and laboratory methods could lead to differences in reported prevalence. The relatively high prevalence observed in this study highlights the importance of routine screening and management of anemia in HF patients. Although the present study reports a lower prevalence than some countries, it nonetheless emphasizes anemia as a prevalent and important comorbidity that may affect prognosis and quality of life of HF.

This study reported various prevalence of anemia across subtypes of HF, showing 26.7% in HFrEF, 50.4% in HFmrEF, and 22.9% in HFpEF. Other studies reported different results: in Swedish patients 41% in HFpEF, 35% in HFmrEF, and 32% in HFpEF [9], in Japan the prevalence rate of anemia was 78% in HFpEF patients with CKD [25], in the United States and Canada 35% in HFpEF [8], and in Thailand 67.6% in HFpEF and 51.2% HFrEF [11]. Our study contributes to the increasing evidence that the prevalence of anemia in HF differs markedly by subtype and country. The unexpectedly elevated rate in HFmrEF calls for additional research, whereas the low anemia rate in HFpEF diverges from global trends, perhaps indicating variations in comorbidities or diagnostic methods. Uniform definitions and expanded multi-center studies may aid in elucidating these disparities.

The prevalence of anemia in this study was most commonly 43% in elderly (Above 65 years). Which was in agreement with a study carried out in Thailand, identified that anemia was more prevalent in older HF patients [11]. The consistent result among various research indicates that older age is a significant risk factor for anemia in HF patients. Acknowledging this connection is vital for healthcare providers as it highlights the necessity of regular screening and focused treatment of anemia in older heart failure patients, individualized treatment (such as iron supplementation and erythropoietin therapy), and collaborative care for this at-risk population. Future research investigating the best treatment approaches for this group should be implemented.

The laboratory investigations revealed the mean of Hb level was 10.680 ± 1.5132 SD. Other studies reported the mean of Hb 9.4 ± 1.8 g/dl in Congo [12], and 10.8 g/dL in females and 11.4 g/dL in males in Saudi Arabia [26]. Variations in mean Hb across populations highlight the influence of regional disparities, as seen in studies showing higher anemia prevalence in rural versus urban areas due to differences in healthcare access [24, 27], as well as the impact of nutritional status and micronutrient deficiencies on Hb levels [22, 28]. These findings underscore that low Hb levels in certain populations, such as HF patients, emphasize the need for targeted interventions, including nutritional support programs, iron supplementation, and improvements in healthcare access and management strategies.

Notably, the findings of this study showed that only 29% of anemic HF patients received anemia management. However, these findings should be interpreted with caution, as the study’s definition of anemia may not fully align with clinical thresholds for initiating treatment and, therefore, does not directly indicate undertreatment without considering clinical guidelines or individual patient contexts. Nonetheless, the relatively low proportion of treated patients may reflect multiple contributing factors, as highlighted by Makubi et al. (2017) in sub-Saharan Africa [23]. These include limited healthcare resources, such as restricted access to diagnostic tools and intravenous iron therapies; lack of standardized treatment protocols, leading to inconsistent clinical practices; and financial constraints, which impede patients’ ability to obtain necessary care [23]. Collectively, these factors underscore the need for comprehensive strategies to improve anemia management among HF patients in resource-limited settings.

Our study has some clinical limitations: (1) its retrospective, cross‑sectional design limits causal inference between anemia and HF progression, (2) essential data of serum ferritin, transferrin saturation, natriuretic peptides, and echocardiographic measures were inconsistently available, preventing us to identify the sub classes of anemia and correlation between anemia severity with cardiac function; (3) reliance on routine medical records may underreport subclinical decompensations and outpatient anemia management, underestimating true prevalence and treatment patterns; and (4) as a single‑center study in Central Sudan, findings may not generalize to regions with different patient demographics or healthcare resources. Additionally, as data for non-anemic HF patients were not collected, direct comparisons between anemic and non-anemic patients could not be performed, highlighting the need for future prospective studies to collect comprehensive data for both groups. Moreover, the external validity of our findings should be interpreted cautiously. While these results provide important baseline insights for Central Sudan, differences in healthcare infrastructure, socioeconomic conditions, and comorbidity profiles may limit generalizability to other regions of Sudan, broader African contexts, or global populations. Nonetheless, in regions with similar health system constraints and nutritional challenges, our data may be particularly relevant and help inform screening and management strategies for anemia in HF.

Despite all these limitations this study has some strengths: (1) a large, well-defined cohort of 557 admissions (266 anemic), ensuring precise prevalence and subgroup insights; (2) use of WHO anemia criteria and a clear HF hospitalization definition for consistent, comparable results; (3) as the first study from Sudan to assess anemia in HF, it fills a critical regional knowledge gap.

## Conclusion

Anemia is common among Sudanese HF patients, with a prevalence of 47.8% in the full cohort and 51% in the analytic cohort with evaluable baseline Hb. It was more prevalent in older adults and patients with HFmrEF. About one-third of HF patients received anemia management, the majority of them received iron supplementation and blood transfusions. These findings emphasize the urgent need for routine anemia screening in HF management protocols, enhanced provider training on anemia HF interplay, improved access to diagnostic tools and treatments like iron supplementation, and future efforts should focus on standardized protocols that combine HF and anemia management.

## Data Availability

No datasets were generated or analysed during the current study.
